# An Efficient Parallel Reverse Conversion of Residue Code to Mixed-Radix Representation Based on the Chinese Remainder Theorem

**DOI:** 10.3390/e24020242

**Published:** 2022-02-05

**Authors:** Mikhail Selianinau, Yuriy Povstenko

**Affiliations:** Department of Mathematics and Computer Sciences, Faculty of Science and Technology, Jan Dlugosz University in Czestochowa, al. Armii Krajowej 13/15, 42-200 Czestochowa, Poland; m.selianinau@ujd.edu.pl

**Keywords:** Residue Number System, modular arithmetic, residue-to-binary conversion, Chinese Remainder Theorem, mixed-radix representation

## Abstract

In this paper, we deal with the critical problems in residue arithmetic. The reverse conversion from a Residue Number System (RNS) to positional notation is a main non-modular operation, and it constitutes a basis of other non-modular procedures used to implement various computational algorithms. We present a novel approach to the parallel reverse conversion from the residue code into a weighted number representation in the Mixed-Radix System (MRS). In our proposed method, the calculation of mixed-radix digits reduces to a parallel summation of the small word-length residues in the independent modular channels corresponding to the primary RNS moduli. The computational complexity of the developed method concerning both required modular addition operations and one-input lookup tables is estimated as $O\left(k^{2}/2\right)$, where *k* equals the number of used moduli. The time complexity is $O\left(\left\lceil \log_{2}k\right\rceil \right)$ modular clock cycles. In pipeline mode, the throughput rate of the proposed algorithm is one reverse conversion in one modular clock cycle.

## 1. Introduction

Along with the improvement of computer technology, the development and implementation of new effective approaches to the organization and realization of computational tasks are some of the main ways to increase the data processing speed. At present, high-performance computing is developing extremely rapidly. These reasons lead to qualitatively new requirements imposed on number-theoretic methods and computational algorithms. Practically, all well-known approaches to high-performance computing use certain parallel forms of data representation and processing. In recent decades, special consideration has been given to the so-called modular computational structures. Their arithmetic foundation is the Residue Number System (RNS), whose ideological roots go back to the classic topics of number theory and abstract algebra. The RNS is a non-positional number system with inherent parallelism and occupies a place of particular importance due to its carry-free properties, which provide a high potential for accelerating arithmetic operations.

As is well known, the RNS has some advantages over a conventional Weighted Number System (WNS) in the design and implementation of high-performance computing applications, devices, and systems. From its appearance in the mid-1950s to the present, RNS arithmetic has attracted the constant attention of researchers in computer technology [1,2], number-theoretic methods [3,4,5], digital signal and image processing [2,5,6,7,8], communications systems [5,9], cryptography [2,8,10,11], and other fields [10].

The main advantage of RNS is its unique ability to decompose the large word-length numbers into a set of smaller word-length residues, which are processed in parallel in the independent modular channels. The inherent parallelism of RNS enables avoiding the carry-overs obtained in addition, subtraction, and multiplication, which are usually time-consuming in the WNS. In this regard, the modularity and carry-free properties make computation fast and efficient. Therefore, the RNS presents one of the most efficient means for increasing data processing speed.

Due to its carry-free property, the residue arithmetic is exceptionally suitable for a broad class of applications in which addition and multiplication are the dominant arithmetic operations. In any case, it has excellent potential for many substantial applications in such areas as digital signal processing, cryptography, distributed information and communication systems, information security systems, fault tolerance, cloud computing, and others. Moreover, these RNS applications may be effectively embedded in processor platforms functioning according to the conventional information-processing approach [2,5,8]. For the reasons mentioned above, residue arithmetic represents an efficient mathematical tool for the high-speed implementation of various computational tasks.

The reverse conversion and base extension are the most critical topics in residue arithmetic. As opposed to conventional WNS, these operations, on a par with other central non-modular procedures such as magnitude comparison, sign determination, overflow detection, general division, scaling, etc., are relatively harder for implementation. They are time consuming and costly due to their more complicated structure compared to modular operations.

As is known, to perform non-modular operations, it is necessary to carry out the binary reconstruction of the integer by its residue code, which in general is hampered by the non-weighted nature of the RNS. This circumstance negates to a substantial extent the main advantages of residue arithmetic.

Therefore, the development of novel approaches and methods for fast number reconstruction by its residue code has significant importance in high-performance computing based on parallel algorithmic structures of RNS, especially for high-speed implementing digital signal processing applications and public-key cryptosystems. That should enable the extensive use of residue arithmetic in many priority areas of science and technology.

In this paper, we present a novel approach to the parallel reverse conversion from the residue code into the mixed-radix representation. In the proposed method, the calculation of mixed-radix digits reduces to a parallel summation of the small word-length residues in the independent modular channels corresponding to the primary RNS moduli.

The paper is structured as follows. Section 2 and Section 3 discuss the basic theoretical concepts of the research. Section 4 describes the mathematical background of the proposed reverse conversion method. Section 5 and Section 6 present a numerical example and an analysis of the computational cost, respectively. Section 7 provides discussion, and Section 8 concludes the paper.

## 2. The Basic Concepts of the Residue Arithmetic

The abstract algebra and number theory create the theoretical basis of the residue arithmetic [12,13].

An RNS is defined by an ordered set $\left\{m_{1},\;m_{2},\cdots ,\;m_{k}\right\}$ of *k* pairwise relatively prime moduli, where each modulus $m_{i}\geq 2$(i=1,2,⋯,k), and the greatest common divisor of $m_{i}$ and $m_{j}$ equals 1, i.e., $\gcd\left(m_{i},m_{j}\right)=1$ for i≠j. For convenience, we assume that the default order of moduli is ascending, i.e., $m_{1}<m_{2}<\cdots <m_{k}$.

In the given RNS, it is possible to represent $M_{k}$ integer numbers, where $M_{k}$ is the product of all moduli, $M_{k}=\prod_{i=1}^{k}m_{k}$. Therefore, the set $Z_{M_{k}}=\left\{0,1,\cdots ,M_{k}-1\right\}$ is usually used as an RNS dynamic range.

Every number $X\in Z_{M_{k}}$ has a unique representation in the form of a *k*-tuple of small integers $\left(\chi_{1},\chi_{2},\cdots ,\chi_{k}\right)$, which is called a residue code, where $\chi_{i}$ is a least non-negative remainder of a division of *X* by $m_{i}$$\left(i=1,2,\cdots ,k\right)$. We can notationally write this relation as $\chi_{i}={\left|X\right|}_{m_{i}}$, where $\chi_{i}\in Z_{m_{i}}=\left\{0,1,\cdots ,m_{i}-1\right\}$.

The main advantage of the residue arithmetic over conventional binary arithmetic consists of parallel carrying out addition, subtraction, and multiplication at the level of small word-length residues. The modular operations $\circ \in \left\{+,-,\times \right\}$ on integers $A=\left(\alpha_{1},\alpha_{2},\cdots ,\alpha_{k}\right)$ and $B=\left(\beta_{1},\beta_{2},\cdots ,\beta_{k}\right)$ are performed independently in each modular channel in compliance with the computational rule:

(1) $$\begin{array}{ll} A & \circ \;B=\left(\alpha_{1},\alpha_{2},\cdots ,\alpha_{k}\right)\circ \left(\beta_{1},\beta_{2},\cdots ,\beta_{k}\right)=\\ & =\left({\left|\alpha_{1}\circ \beta_{1}\right|}_{m_{1}},{\left|\alpha_{2}\circ \beta_{2}\right|}_{m_{2}},\cdots ,{\left|\alpha_{k}\circ \beta_{k}\right|}_{m_{k}}\right), \end{array}$$

where $\alpha_{i}={\left|A\right|}_{m_{i}}$ and $\beta_{i}={\left|B\right|}_{m_{i}},i=1,2,\cdots ,k$.

In other words, the arithmetic operations on long-word operands are decomposed into modular channels with operands that are no larger than the corresponding modulus. Moreover, all the modular channels are entirely independent of each other. The carry-free nature of modular operations (1) is one of the most attractive features of residue arithmetic [1,3,8].

Therefore, compared with the conventional WNS, the RNS simplifies and speeds up the addition and multiplication operations. This fundamental advantage of the residue arithmetic strongly appears in the case of implementing computational procedures, which mainly contain long segments consisting of only sequences of modular arithmetic operations. In this case, the primary moduli set is chosen so that the final results of the computational procedure always belong to the used dynamic range for any allowed values of input operands. At the same time, the intermediate results can even exceed the boundaries of the dynamic range.

Along with the carry-free modular operations, there are also the so-called non-modular operations such as residue-to-binary conversion, base extension, magnitude comparison, sign determination, overflow detection, general division, scaling, etc. These operations are complicated and quite time consuming, and their significant computational complexity limits the applications of the residue arithmetic and restricts its widespread usage for high-speed computing.

To perform the non-modular operations, it is required to consider all residues in the *k*-tuple $\left(\chi_{1},\chi_{2},\cdots ,\chi_{k}\right)$. Furthermore, it is necessary to determine the integer value of the number by its residue code, which in general is hampered by the non-positional nature of the RNS. The crucial problem of efficient implementation of non-modular operations is constantly receiving considerable attention by modern researchers [2,5,8].

The applicability of residue arithmetic is mainly determined by the computational complexity and feasibility of non-modular operations, which are used as a basis for implementing more complex computational algorithms in RNS. At the same time, the fundamental problem in the residue arithmetic, which unfortunately up to now is yet completely unresolved; it consists of reducing the computational complexity of non-modular operations. Due to a lack of efficient methods and algorithms for non-modular operations implementation, the residue arithmetic is mainly suitable when the modular additions and multiplications make up the bulk of required computations. In this case, the number of used non-modular operations is relatively small. This circumstance bounds the widespread use of the RNS to a narrow class of specific tasks.

## 3. Reverse Conversion of the Residue Code to Conventional Representation

The root problem of residue arithmetic is that the weighted value of the integer *X* depends on all the residues $\chi_{1},\chi_{2},\cdots ,\chi_{k}$. The reconstruction of an integer by its residue code, i.e., the reverse conversion, is one of the most difficult non-modular operations in residue arithmetic. Moreover, this operation underlies all the other non-modular procedures.

Despite the currently extensive studies on residue arithmetic and its applications, there is a need to develop novel efficient approaches and methods of an integer number reconstruction by its residue code. This should enable us the extensive use of residue arithmetic for high-speed computing in many priority fields, first of all, in various digital signal processing and cryptographic applications.

There are two canonical techniques of reverse conversion: the canonical method based on the Chinese Remainder Theorem (CRT) and the residue code conversion to a weighted representation in the Mixed-Radix System (MRS) [1,2,5,8,14,15,16,17,18]. In general, all other conversion methods represent different variants of these two methods.

Below, we describe the mathematical background of these methods.

### 3.1. CRT-Base Conversion Method

When the moduli $m_{1},m_{2},\cdots ,m_{k}$ are pairwise relatively prime, the integer number *X* and its residue code $\left(\chi_{1},\chi_{2},\cdots ,\chi_{k}\right)$ are related by the equation:

(2) $$X={\left|\sum_{i=1}^{k}M_{i,k}\chi_{i,k}\right|}_{M_{k}},$$

where $M_{i,k}=M_{k}/m_{i}$, $\chi_{i,k}={\left|M_{i,k}^{-1}\chi_{i}\right|}_{m_{i}}$ is a normalized residue modulo $m_{i}$$\left(i=1,2,\cdots ,k\right)$, ${\left|Y^{-1}\right|}_{m}$ denotes the multiplicative inverse of an integer *Y* modulo *m*.

In essence, Equation (2) represents the CRT [10,19,20].

In the last decades, considerable efforts are directed to reducing the complexity of the CRT implementation and the possibility of its application in high-speed computing [2,5,8,21,22,23]. The main idea of these methods is to replace the inner multiplications and additions modulo $M_{k}$ with simpler operations (see (2)).

Consider the CRT-number

(3) $$X_{k}=\sum_{i=1}^{k}M_{i,k}\chi_{i,k}.$$

As follows from (2), the difference $X_{k}-X$ is a multiple of $M_{k}$. Therefore, the following exact integer equality holds

(4) $$X=X_{k}-\rho_{k}\left(X\right)M_{k}.$$

The unique integer number $\rho_{k}\left(X\right)$ is a normalized rank (or, briefly, rank) of the number *X* [3,4,7].

Equation (4) is called a rank form of the integer *X*. In essence, the rank $\rho_{k}\left(X\right)$ is a reconstruction coefficient that indicates how many times the dynamic range $M_{k}$ is exceeded when converting the residue code $\left(\chi_{1},\chi_{2},\cdots ,\chi_{k}\right)$ to the integer *X*.

In contrast to (2), Equation (4) does not contain a very time-consuming reduction modulo $M_{k}$. Therefore, when we have the efficient method for the rank $\rho_{k}\left(X\right)$ computation, the reverse conversion algorithm constructed on the basis of (4) has a substantial lead over the canonical CRT implementation (2).

### 3.2. MRS-Base Conversion Method

In the MRS defined by a set $\left\{m_{1},m_{2},\cdots ,m_{k}\right\}$ of pairwise relatively prime moduli, the integer $X\in Z_{M_{k}}$ is represented by the *k*-tuple $\left(x_{k},x_{k-1},\cdots ,x_{1}\right)$ of mixed-radix digits, resulting in

(5) $$X=x_{1}+x_{2}M_{1}+x_{3}M_{2}+\cdots +x_{k}M_{k-1}=\sum_{i=1}^{k}x_{i}M_{i-1},$$

where $x_{i}\in Z_{m_{i}}$(i=1,2,⋯,k) [1,2,8].

It is well known that the MRS surpasses the RNS when performing non-modular operations such as magnitude comparison, sign determination, and overflow detection. Therefore, the mixed-radix representation has received the widest appliance for the implementation of non-modular procedures along with the other generally accepted integral characteristics of the residue code such as the rank of a number, core function, interval index, parity function, diagonal, and quotient functions [3,4,7,24,25,26,27,28,29,30,31,32,33].

The RNS-to-MRS reverse conversion establishes an association between the residue code $\left(\chi_{1},\chi_{2},\cdots ,\chi_{k}\right)$ of the number *X* and its mixed-radix representation $\left(x_{k},x_{k-1},\cdots ,x_{1}\right)$. The mixed-radix digits $x_{i}$(i=1,2,⋯,k) in (5) are computed according to the following calculation relations [1]:

$$\begin{array}{c} x_{1}=\chi_{1}, \end{array}$$

$$\begin{array}{c} x_{2}={\left|\left(\chi_{2}-x_{1}\right){\left|m_{1}^{-1}\right|}_{m_{2}}\right|}_{m_{2}}, \end{array}$$

$$\begin{array}{c} x_{3}={\left|\left(\left(\chi_{3}-x_{1}\right){\left|m_{1}^{-1}\right|}_{m_{3}}-x_{2}\right){\left|m_{2}^{-1}\right|}_{m_{3}}\right|}_{m_{3}}, \end{array}$$

$$\begin{array}{c} \cdots \end{array}$$

$$\begin{array}{c} x_{k}={\left|\left(\cdots \left(\left(\chi_{k}-x_{1}\right){\left|m_{1}^{-1}\right|}_{m_{k}}-x_{2}\right){\left|m_{2}^{-1}\right|}_{m_{k}}-\cdots -x_{k-1}\right){\left|m_{k-1}^{-1}\right|}_{m_{k}}\right|}_{m_{k}}. \end{array}$$

This sequential calculation procedure called a chained algorithm can be written in the general form

(6) $$x_{i}={\left|X^{\left(i\right)}\right|}_{m_{i}},$$

where

(7) $$X^{\left(i\right)}=\left\{\begin{array}{c} X,\mathrm{if}\;i=1,\\ \left(X^{\left(i-1\right)}-x_{i-1}\right)m_{i-1}^{-1},\mathrm{if}\;i=2,3,\cdots ,k. \end{array}\right.$$

From (6) and (7), it follows that the considered computational process requires two modular operations: subtraction and multiplication by the multiplicative inverse. Thus, the most crucial advantage of this algorithm is its high modularity. However, its strictly sequential nature prevents general use for the construction of appropriate high-performance parallel computing procedures.

## 4. A Novel CRT-Base RNS-to-MRS Reverse Conversion Method

Now, we describe a proposed new method for calculating mixed-radix digits $x_{1},x_{2},\cdots ,x_{k}$ of the number *X* by its residue code $\left(\chi_{1},\chi_{2},\cdots ,\chi_{k}\right)$.

Consider the CRT-number $X_{k}$. According to (3), we have

(8) $$X_{k}=\sum_{i=1}^{k-1}M_{i,k-1}m_{k}\chi_{i,k}+M_{k-1}\chi_{k,k}.$$

By Euclid’s Division Lemma, the integer $m_{k}\chi_{i,k}$ can be written as

(9) $$m_{k}\chi_{i,k}=\chi_{i,k-1}+\left\lfloor \frac{m_{k}\chi_{i,k}}{m_{i}}\right\rfloor m_{i},$$

where

$$\begin{array}{c} \chi_{i,k-1}={\left|m_{k}\chi_{i,k}\right|}_{m_{i}}={\left|m_{k}{\left|M_{i,k}^{-1}\chi_{i}\right|}_{m_{i}}\right|}_{m_{i}}={\left|m_{k}M_{i,k}^{-1}\chi_{i}\right|}_{m_{i}}={\left|M_{i,k-1}^{-1}\chi_{i}\right|}_{m_{i}}, \end{array}$$

$\left\lfloor x\right\rfloor$ denotes the largest integer less than or equal to *x*.

Substituting (9) into (8), we obtain

(10) $$X_{k}=X_{k-1}+M_{k-1}S_{k}\left(X\right),$$

where

(11) $$X_{k-1}=\sum_{i=1}^{k-1}M_{i,k-1}\chi_{i,k-1},$$

(12) $$S_{k}\left(X\right)=\sum_{i=1}^{k}R_{i,k}\left(\chi_{i}\right),$$

(13) $$R_{i,k}\left(\chi_{i}\right)=\left\lfloor \frac{m_{k}\chi_{i,k}}{m_{i}}\right\rfloor \;\left(i=1,2,\cdots ,k\right).$$

Taking into account (9), we have

$$\begin{array}{c} R_{i,k}\left(\chi_{i}\right)=\frac{m_{k}\chi_{i,k}-\chi_{i,k-1}}{m_{i}}. \end{array}$$

Since $R_{i,k}\left(\chi_{i}\right)\in Z_{m_{k}}$, we can reduce the right side of equality modulo $m_{k}$.

Hence, the residue $R_{i,k}\left(\chi_{i}\right)$ can be calculated as

(14) $$R_{i,k}\left(\chi_{i}\right)={\left|-\frac{\chi_{i,k-1}}{m_{i}}\right|}_{m_{k}}={\left|-\frac{{\left|M_{i,k-1}^{-1}\chi_{i}\right|}_{m_{i}}}{m_{i}}\right|}_{m_{k}}\left(i=1,2,\cdots ,k-1\right).$$

At the same time, from (13) it follows that

(15) $$R_{k,k}\left(\chi_{k}\right)=\chi_{k,k}={\left|M_{k,k}^{-1}\chi_{k}\right|}_{m_{k}}={\left|M_{k-1}^{-1}\chi_{k}\right|}_{m_{k}}.$$

Similarly, taking into account Equations (10)–(13), the numbers $X_{i}$(i=k−1,k−2,⋯,1) can be written by turns as

$$\begin{array}{c} X_{k-1}=X_{k-2}+M_{k-2}S_{k-1}\left(X\right), \end{array}$$

$$\begin{array}{c} X_{k-2}=X_{k-3}+M_{k-3}S_{k-2}\left(X\right), \end{array}$$

$$\begin{array}{c} \cdots \end{array}$$

$$\begin{array}{c} X_{2}=X_{1}+M_{1}S_{2}\left(X\right), \end{array}$$

$$\begin{array}{c} X_{1}=M_{0}S_{1}\left(X\right), \end{array}$$

where $M_{0}=1$, $S_{1}\left(X\right)=\chi_{1}$, the integers $S_{l}\left(X\right)$$\left(l=2,3,\cdots ,k\right)$ are calculated according to (12)–(15) in the case when the index *k* is replaced by *l*.

Finally, substituting the above equations for $X_{l}$(l=k−1,k−2,⋯,1) by turns into (10), we obtain

(16) $$X_{k}=\sum_{i=1}^{k}M_{l-1}S_{l}\left(X\right).$$

At the same time, according to Euclid’s Division Lemma, we have

(17) $$S_{l}\left(X\right)=R_{l}\left(X\right)+m_{l}Q_{l}\left(X\right),$$

where $R_{l}\left(X\right)={\left|S_{l}\left(X\right)\right|}_{m_{l}}$ and $Q_{l}\left(X\right)=\left\lfloor S_{l}\left(X\right)/m_{i}\right\rfloor$ are the remainder and quotient of the division $S_{l}\left(X\right)$ by the modulus $m_{l}$, respectively.

Therefore, taking into account (12), when the index *k* is replaced by *l*, the integers $R_{l}\left(X\right)$ and $Q_{l}\left(X\right)$ can be computed as

(18) $$R_{l}\left(X\right)={\left|\sum_{i=1}^{l}R_{i,l}\left(\chi_{i}\right)\right|}_{m_{l}},$$

(19) $$Q_{l}\left(X\right)=\left\lfloor \frac{1}{m_{l}}\sum_{i=1}^{l}R_{i,l}\left(\chi_{i}\right)\right\rfloor .$$

From (19), it follows that $Q_{l}\left(X\right)$ equals the number of occurred overflows when calculating the sum $R_{l}\left(X\right)$ of residues $R_{1,l}\left(\chi_{1}\right),R_{2,l}\left(\chi_{2}\right),\cdots ,R_{l,l}\left(\chi_{l}\right)$ modulo $m_{l}$$\left(l=2,3,\cdots ,k\right)$.

Note that $R_{1}\left(X\right)=\chi_{1}$ and $Q_{1}\left(X\right)=0$ since $S_{1}\left(X\right)=\chi_{1}$.

Substituting (17) into (16), we obtain

(20) $$X_{k}=X_{k}^{\left(R\right)}+X_{k-1}^{\left(Q\right)}+M_{k}Q_{k}\left(X\right),$$

where

(21) $$X_{k}^{\left(R\right)}=\sum_{l=1}^{k}M_{l-1}R_{l}\left(X\right),$$

(22) $$X_{k-1}^{\left(Q\right)}=\sum_{l=1}^{k-1}M_{l}Q_{l}\left(X\right).$$

Let us draw attention to Equations (21) and (22). It is evident that the number $X_{k}^{\left(R\right)}$ is represented by the *k*-tuple $\left(x_{k}^{\left(R\right)},x_{k-1}^{\left(R\right)},\cdots ,x_{1}^{\left(R\right)}\right)$ of mixed-radix digits, where $x_{l}^{\left(R\right)}=R_{l}\left(X\right)$, l=1,2,⋯,k (see Equation (5)). At the same time, $x_{l}^{\left(R\right)}\in Z_{m_{l}}$ and $X_{k}^{\left(R\right)}\leq M_{k}-1$.

Bearing in mind that $Q_{1}\left(X\right)=0$, the number $X_{k-1}^{\left(Q\right)}$ can be written as

(23) $$X_{k-1}^{\left(Q\right)}=\sum_{l=1}^{k-1}M_{l-1}Q_{l}^{{}^{\prime }}\left(X\right),$$

where $Q_{1}^{{}^{\prime }}\left(X\right)=0$, $Q_{2}^{{}^{\prime }}\left(X\right)=Q_{1}\left(X\right)=0$, and $Q_{l}^{{}^{\prime }}\left(X\right)=Q_{l-1}\left(X\right)$ for l≥3. Therefore, taking into account (19), the integer $Q_{l}^{{}^{\prime }}\left(X\right)$ can be calculated as

(24) $$Q_{l}^{{}^{\prime }}\left(X\right)=\left\lfloor \frac{1}{m_{l-1}}\sum_{i=1}^{l-1}R_{i,l-1}\left(\chi_{i}\right)\right\rfloor \;\left(l=3,4,\cdots ,k\right).$$

Hence, $Q_{l}^{{}^{\prime }}\left(X\right)<l-1$ since $R_{i,l-1}\left(\chi_{i}\right)\leq m_{l-1}-1$.

Thus, the integer $X_{k-1}^{\left(Q\right)}$ (see Equations (23) and (5)) can be represented by a *k*-tuple $\left(x_{k}^{\left(Q\right)},x_{k-1}^{\left(Q\right)},\cdots ,x_{1}^{\left(Q\right)}\right)$ of mixed-radix digits under the condition that $x_{l}^{\left(Q\right)}\in Z_{m_{l}}\left(l=1,2,\cdots ,k\right)$, where $x_{1}^{\left(Q\right)}=x_{2}^{\left(Q\right)}=0,x_{l}^{\left(Q\right)}=Q_{l}^{{}^{\prime }}\left(X\right)$ for l>2. Consequently, that entails the fulfillment of the condition $Z_{l-1}\subset Z_{m_{l}}$, which leads to inequality

(25) $$m_{l}\geq l-1\;\left(l=1,2,\cdots ,k\right).$$

Thus, when the moduli set $\left\{m_{1},m_{2},\cdots ,m_{k}\right\}$ meets the conditions (25), we have that $X_{k-1}^{\left(Q\right)}<M_{k}$.

Note that the integer $X_{k-1}^{\left(Q\right)}$ is a multiple of the number $M_{2}=m_{1}m_{2}$ because of $x_{1}^{\left(Q\right)}=x_{2}^{\left(Q\right)}=0$ (see Equation (5)).

Now, let us return to Equation (20). According to Euclid’s Division Lemma, the sum of two mixed-radix numbers $X_{k}^{\left(R\right)}$ and $X_{k-1}^{\left(Q\right)}$ results in

(26) $$X_{k}^{\left(R\right)}+X_{k-1}^{\left(Q\right)}={\left|X_{k}^{\left(R\right)}+X_{k-1}^{\left(Q\right)}\right|}_{M_{k}}+M_{k}\left\lfloor \frac{X_{k}^{\left(R\right)}+X_{k-1}^{\left(Q\right)}}{M_{k}}\right\rfloor .$$

Hence, substituting (26) into (20), we obtain

(27) $$X_{k}={\left|X_{k}^{\left(R\right)}+X_{k-1}^{\left(Q\right)}\right|}_{M_{k}}+M_{k}\left(Q_{k}\left(X\right)+\left\lfloor \frac{X_{k}^{\left(R\right)}+X_{k-1}^{\left(Q\right)}}{M_{k}}\right\rfloor \right).$$

Taking into account the rank form of the number *X* (4), from (27) we have

(28) $$X={\left|X_{k}^{\left(R\right)}+X_{k-1}^{\left(Q\right)}\right|}_{M_{k}}.$$

From (28), it follows that the mixed-radix representation of the number *X*, i.e., *k*-tuple $\left(x_{k},x_{k-1},\cdots ,x_{1}\right)$, can be calculated as a result of the addition of two mixed-radix numbers $X_{k}^{\left(R\right)}=\left(x_{k}^{\left(R\right)},x_{k-1}^{\left(R\right)},\cdots ,x_{1}^{\left(R\right)}\right)$ and $X_{k-1}^{\left(Q\right)}=\left(x_{k}^{\left(Q\right)},x_{k-1}^{\left(Q\right)},\cdots ,x_{1}^{\left(Q\right)}\right)$ (see (21) and (23)) in the basis $\left\{m_{1},m_{2},\cdots ,m_{k}\right\}$. Note that $x_{1}^{\left(R\right)}=\chi_{1}$, $x_{1}^{\left(Q\right)}=x_{2}^{\left(Q\right)}=0$. At the same time, the digits $x_{2}^{\left(R\right)},x_{3}^{\left(R\right)},\cdots ,x_{k}^{\left(R\right)}$ and $x_{3}^{\left(Q\right)},x_{4}^{\left(Q\right)},\cdots ,x_{k}^{\left(Q\right)}$ are calculated as the sum of the residues $R_{1,l}\left(\chi_{1}\right),R_{2,l}\left(\chi_{2}\right),\cdots ,R_{l,l}\left(\chi_{l}\right)$ modulo $m_{l}$ along with the counting of occurred overflows according to (18) and (24) $\left(l=2,3,\cdots ,k\right)$.

Therefore, the mixed-radix digits $x_{l}^{\left(R\right)}$ and $x_{l}^{\left(Q\right)}$ are computed as

(29) $$x_{1}^{\left(R\right)}=\chi_{1},\;\;x_{l}^{\left(R\right)}={\left|\sum_{i=1}^{l}R_{i,l}\left(\chi_{i}\right)\right|}_{m_{l}}\;\left(l=2,3,\cdots ,k\right),$$

(30) $$x_{1}^{\left(Q\right)}=x_{2}^{\left(Q\right)}=0,\;\;x_{l}^{\left(Q\right)}=\left\lfloor \frac{1}{m_{l-1}}\sum_{i=1}^{l-1}R_{i,l-1}\left(\chi_{i}\right)\right\rfloor \;\left(l=3,4,\cdots ,k\right),$$

where

(31) $$R_{i,l}\left(\chi_{i}\right)={\left|-\frac{{\left|M_{i,l-1}^{-1}\chi_{i}\right|}_{m_{i}}}{m_{i}}\right|}_{m_{l}}\;\left(i\neq l\right),$$

(32) $$R_{l,l}\left(\chi_{l}\right)={\left|M_{l-1}^{-1}\chi_{l}\right|}_{m_{l}}\;\left(l=2,3,\cdots ,k\right).$$

Furthermore, in the MRS with the bases $m_{1},m_{2},\cdots ,m_{k}$, we calculate the sum of two numbers $X_{k}^{\left(R\right)}$ and $X_{k-1}^{\left(Q\right)}$. As a result, we obtain the mixed-radix representation $\left(x_{k},x_{k-1},\cdots ,x_{1}\right)$ of the number *X*.

Table 1 given below presents the pre-calculation components (see Equations (31) and (32)). It should be recalled that $\langle R_{1,1}\left(\chi_{1}\right)\rangle =\chi_{1}$. The abbreviation LUT means lookup table. The bit-length of residues is $b_{l}=\left\lceil \log_{2}m_{l}\right\rceil$$\left(l=1,2,\cdots ,k\right)$. Here, and further, $\left\lceil x\right\rceil$ denotes the smallest integer greater than or equal to *x*.

Table 2 presents the results of calculations in the modular channels according to Equations (29) and (30). It should be reminded that in the first modular channel corresponding to the modulus $m_{1}$, the calculations are not carried out, so $x_{1}^{\left(R\right)}=\chi_{1}$ and $x_{2}^{\left(Q\right)}=0$.

The stated above allows us to formulate the following substantial theorem.

**Theorem** **1.** **(About RNS-to-MRS reverse conversion)**.

*Let an arbitrary RNS be defined by an ascending-ordered set of k pairwise relatively prime moduli $m_{1},m_{2},\cdots ,m_{k}$ ($m_{l}\geq l-1$, l=1,2,⋯,k, k≥2), and let the residue code $\left(\chi_{1},\chi_{2},\cdots ,\chi_{k}\right)$ of the number $X\in Z_{M_{k}}$ be given. Then, the mixed-radix representation $\left(x_{k},x_{k-1},\cdots ,x_{1}\right)$ of the number X can be computed as a result of the summation of two mixed-radix numbers, namely, the appropriate number $X_{k}^{\left(R\right)}=\left(x_{k}^{\left(R\right)},x_{k-1}^{\left(R\right)},\cdots ,x_{1}^{\left(R\right)}\right)$ and the correction number $X_{k-1}^{\left(Q\right)}=\left(x_{k}^{\left(Q\right)},x_{k-1}^{\left(Q\right)},\cdots ,x_{1}^{\left(Q\right)}\right)$, where the digits $x_{l}^{\left(R\right)}$ and $x_{l}^{\left(Q\right)}$$\left(l=1,2,\cdots ,k\right)$ are calculated according to (29) and (30), respectively, taking into account (31) and (32).*

## 5. A Numerical Example of the Proposed Conversion Method

The main idea of the proposed approach to reverse conversion is illustrated below by a simple numerical example. For convenience, we consider a four-moduli RNS.

**Example** **1.** *Let the RNS moduli-set be $\left\{m_{1},m_{2},m_{3},m_{4}\right\}$ = $\left\{5,7,9,11\right\}$. Suppose that we wish to calculate the digits of the mixed-radix representation $\left(x_{4},x_{3},x_{2},x_{1}\right)$ of the given number X by its residue code $\left(\chi_{1},\chi_{2},\chi_{3},\chi_{4}\right)=\left(3,6,4,2\right)$.*

**Step 1** *. The calculation of the primitive constants in a given RNS.*

$M_{4}=3465,\;M_{3}=315,\;M_{2}=35,\;M_{1}=5,\;M_{0}=1,$

$M_{1,4}=693,\;M_{2,4}=495,\;M_{3,4}=385,\;M_{4,4}=315,$

${\left|M_{1,4}^{-1}\right|}_{m_{1}}=2,\;{\left|M_{2,4}^{-1}\right|}_{m_{2}}=3,\;{\left|M_{3,4}^{-1}\right|}_{m_{3}}=4,\;{\left|M_{4,4}^{-1}\right|}_{m_{4}}=8,$

${\left|m_{1}^{-1}\right|}_{m_{4}}=9,\;{\left|m_{2}^{-1}\right|}_{m_{4}}=8,\;{\left|m_{3}^{-1}\right|}_{m_{4}}=5,\;{\left|M_{3}^{-1}\right|}_{m_{4}}=8,$

$M_{1,3}=63,\;M_{2,3}=45,\;M_{3,3}=35,$

${\left|M_{1,3}^{-1}\right|}_{m_{1}}=2,\;{\left|M_{2,3}^{-1}\right|}_{m_{2}}=5,\;{\left|M_{3,3}^{-1}\right|}_{m_{3}}=8,$

${\left|m_{1}^{-1}\right|}_{m_{3}}=2,\;{\left|m_{2}^{-1}\right|}_{m_{3}}=4,\;{\left|M_{2}^{-1}\right|}_{m_{3}}=8,$

$M_{1,2}=7,\;M_{2,2}=5,$

${\left|M_{1,2}^{-1}\right|}_{m_{1}}=3,\;{\left|M_{2,2}^{-1}\right|}_{m_{2}}=3,$

${\left|m_{1}^{-1}\right|}_{m_{2}}=3,\;{\left|M_{1}^{-1}\right|}_{m_{2}}=3.$

**Step 2** *. The calculation of the residue sets $\langle R_{1,l}\left(\chi_{1}\right),R_{2,l}\left(\chi_{2}\right),\cdots ,R_{l,l}\left(\chi_{l}\right)\rangle$ according to (31) and (32) $\left(l=1,2,3,4\right)$.*

*We obtain*

$R_{1,1}\left(\chi_{1}\right)=\chi_{1}=3,$

$R_{1,2}\left(\chi_{1}\right)={\left|-{\left|1\cdot 3\right|}_{5}\cdot 3\right|}_{7}=5,$

$R_{2,2}\left(\chi_{2}\right)={\left|3\cdot 6\right|}_{7}=4,$

$R_{1,3}\left(\chi_{1}\right)={\left|-{\left|3\cdot 3\right|}_{5}\cdot 2\right|}_{9}=1,$

$R_{2,3}\left(\chi_{2}\right)={\left|-{\left|3\cdot 6\right|}_{7}\cdot 4\right|}_{9}=2,$

$R_{3,3}\left(\chi_{3}\right)={\left|8\cdot 4\right|}_{9}=5,$

$R_{1,4}\left(\chi_{1}\right)={\left|-{\left|2\cdot 3\right|}_{5}\cdot 9\right|}_{11}=2,$

$R_{2,4}\left(\chi_{2}\right)={\left|-{\left|5\cdot 6\right|}_{7}\cdot 8\right|}_{11}=6,$

$R_{3,4}\left(\chi_{3}\right)={\left|-{\left|8\cdot 4\right|}_{9}\cdot 5\right|}_{11}=8,$

$R_{4,4}\left(\chi_{4}\right)={\left|8\cdot 2\right|}_{11}=5.$

*As a result, the following sets of residues occur*

$\langle R_{1,1}\left(\chi_{1}\right)\rangle =\langle 3\rangle ,$

$\langle R_{1,2}\left(\chi_{1}\right),\;R_{2,2}\left(\chi_{2}\right)\rangle =\langle 5,4\rangle ,$

$\langle R_{1,3}\left(\chi_{1}\right),\;R_{2,3}\left(\chi_{2}\right),\;R_{3,3}\left(\chi_{3}\right)\rangle =\langle 1,2,5\rangle ,$

$\langle R_{1,4}\left(\chi_{1}\right),\;R_{2,4}\left(\chi_{2}\right),\;R_{3,4}\left(\chi_{3}\right),\;R_{4,4}\left(\chi_{4}\right)\rangle =\langle 2,6,8,5\rangle .$

**Step 3.** *The summation of the residues $R_{1,l}\left(\chi_{1}\right),\;R_{2,l}\left(\chi_{2}\right),\cdots ,R_{l,l}\left(\chi_{l}\right)$ modulo $m_{l}$ along with the counting of occurring overflows according to (18) and (19), respectively $\left(l=2,3,4\right)$.*

*Recall that $R_{1}\left(X\right)=R_{1,1}\left(\chi_{1}\right)=3$, and $Q_{1}\left(X\right)=0$. We have*

$R_{2}\left(X\right)={\left|5+4\right|}_{7}={\left|9\right|}_{7}=2,$

$R_{3}\left(X\right)={\left|1+2+5\right|}_{9}={\left|8\right|}_{9}=8,$

$R_{4}\left(X\right)={\left|2+6+8+5\right|}_{11}={\left|21\right|}_{11}=10,$

$Q_{2}\left(X\right)=\left\lfloor \left(5+4\right)/7\right\rfloor =\left\lfloor 9/7\right\rfloor =1,$

$Q_{3}\left(X\right)=\left\lfloor \left(1+2+5\right)/9\right\rfloor =\left\lfloor 8/9\right\rfloor =0,$

$Q_{4}\left(X\right)=\left\lfloor \left(2+6+8+5\right)/11\right\rfloor =\left\lfloor 21/11\right\rfloor =1.$

*Therefore, the mixed-radix representations of the numbers $X_{4}^{\left(R\right)}$ and $X_{3}^{\left(Q\right)}$ (see (21) and (23)) are computed:*

$\left(x_{4}^{\left(R\right)},x_{3}^{\left(R\right)},x_{2}^{\left(R\right)},x_{1}^{\left(R\right)}\right)=\left(R_{4}\left(X\right),R_{3}\left(X\right),R_{2}\left(X\right),R_{1}\left(X\right)\right)=\left(10,8,2,3\right),$

*$\left(x_{4}^{\left(Q\right)},x_{3}^{\left(Q\right)},x_{2}^{\left(Q\right)},x_{1}^{\left(Q\right)}\right)=\left(Q_{3}\left(X\right),Q_{2}\left(X\right),0,0\right)=\left(0,1,0,0\right)$.*

**Step 4.** *The calculation of the mixed-radix digits $\left(x_{4},x_{3},x_{2},x_{1}\right)$.*

*The addition of two numbers $X_{4}^{\left(R\right)}=\left(10,8,2,3\right)$ and $X_{3}^{\left(Q\right)}=\left(0,1,0,0\right)$ according to (28) gives the mixed-radix representation $\left(0,0,2,3\right)$ of the number X.*

*Let us now verify the obtained result. According to (5), we have*

$$X=\left(0,0,2,3\right)=0\cdot 315+0\cdot 35+2\cdot 5+3=13.$$

*This result holds because the residue code of the integer number X=13 is 3,6,4,2, since ${\left|13\right|}_{5}=3$, ${\left|13\right|}_{7}=6$, ${\left|13\right|}_{9}=4$, ${\left|13\right|}_{11}=2$. Thus, this result coincides with the condition of the example.*

## 6. The Computational Cost of the Reverse Conversion Method

As it follows from the results mentioned above, the calculation of the mixed-radix digits $x_{1}$, $x_{2}$, ⋯, $x_{k}$ reduces to the independent and parallel summation of small residues $R_{1,l}\left(\chi_{1}\right)$, $R_{2,l}\left(\chi_{2}\right)$, ⋯, $R_{l,l}\left(\chi_{l}\right)$ modulo $m_{l}$ in *l*th modular channel $\left(l=1,2,\cdots ,k\right)$, taking into account the number of the overflows occuring during the modular addition operations (see (29)–(32)).

Let us evaluate the time required to perform the parallel reverse conversion.

First, we consider the calculation of mixed-radix digits of the numbers $X_{k}^{\left(R\right)}=\left(x_{k}^{\left(R\right)},x_{k-1}^{\left(R\right)},\cdots ,x_{1}^{\left(R\right)}\right)$ and $X_{k-1}^{\left(Q\right)}=\left(x_{k}^{\left(Q\right)},x_{k-1}^{\left(Q\right)},\cdots ,x_{1}^{\left(Q\right)}\right)$ (see (29) and (30)). As can be seen, there are no modular addition operations in the first modular channel corresponding to the modulus $m_{1}$. In the second channel, we have only one addition operation modulo $m_{2}$. Furthermore, two additions modulo $m_{3}$ are performed in the third channel and so on. Thus, in the *l*th modular channel, we have l−1 additions modulo $m_{l}$$\left(l=2,3,\cdots ,k\right)$. These calculations are easily parallelized and pipelined. Therefore, the required computation time for calculating digits $x_{l}^{\left(R\right)}$ and $x_{l}^{\left(Q\right)}$ is $T_{l}=\left\lceil \log_{2}l\right\rceil$ modular clock cycles.

Thus, the time for obtaining the mixed-radix representations of the numbers $X_{k}^{\left(R\right)}$ and $X_{k-1}^{\left(Q\right)}$ is determined by the time in the *k*th modular channel and equals $T_{k}=\left\lceil \log_{2}k\right\rceil$ modular clock cycles.

The summation of $X_{k}^{\left(R\right)}$ and $X_{k-1}^{\left(Q\right)}$ on the bases $\left\{m_{1},m_{2},\cdots ,m_{k}\right\}$ involves two additional modular clock cycles taking into account the inter-digit carries. Therefore, the execution time of the reverse conversion equals $T_{conv}=T_{k}+2$ modular clock cycles. Thus, the overall time is $t_{conv}=T_{conv}t_{mod}$, where $t_{mod}$ denotes the modular clock cycle time. At the same time, when pipelined, the throughput rate of the proposed conversion method is one conversion in one modular clock cycle.

Consider now the evaluation of the required computational cost. Due to the small word-length of residues in the *k*-tuple $\left(\chi_{1},\chi_{2},\cdots ,\chi_{k}\right)$, the pre-computation and lookup table techniques are suitable for reverse conversion implementation. So, we can use one-input lookup tables depending on the residues word-length in each modular channel.

At the beginning stage of the reverse conversion, in the *l*th channel corresponding to the modulus $m_{l}$, the number of lookup tables required to store the residue set $\langle R_{1,l}\left(\chi_{1}\right),R_{2,l}\left(\chi_{2}\right),\cdots ,R_{l,l}\left(\chi_{l}\right)\rangle$ equals $N_{lut}\left(l\right)=l$. At the same time, the word length of recorded residues is $b_{l}=\left\lceil \log_{2}m_{l}\right\rceil$ bits $\left(l=2,3,\cdots ,k\right)$. In the first modular channel, $N_{lut}\left(1\right)=0$ since $S_{1}\left(X\right)=\chi_{1}$.

Then, the overall number of one-input lookup tables in all modular channels is equal to

$$\begin{array}{c} N_{lut}=\sum_{l=2}^{k}N_{lut}\left(l\right)=\frac{k^{2}+k-2}{2}. \end{array}$$

The summation of the residues $R_{1,l}\left(\chi_{1}\right),R_{2,l}\left(\chi_{2}\right),\cdots ,R_{l,l}\left(\chi_{l}\right)$ modulo $m_{l}$ requires $N_{add}\left(l\right)=l-1$ modular addition operations $\left(l=2,3,\cdots ,k\right)$. At the same time, all independent calculations are realized in parallel in corresponding modular channels.

Taking into account that $x_{1}^{\left(Q\right)}=x_{2}^{\left(Q\right)}=0$, the summation of two numbers $X_{k}^{\left(R\right)}=\left(x_{k}^{\left(R\right)},x_{k-1}^{\left(R\right)},\cdots ,x_{1}^{\left(R\right)}\right)$ and $X_{k-1}^{\left(Q\right)}=\left(x_{k}^{\left(Q\right)},x_{k-1}^{\left(Q\right)},\cdots ,x_{1}^{\left(Q\right)}\right)$ on the final stage of the reverse conversion requires $2\left(k-2\right)$ modular addition operations.

Hence, the overall number of modular addition operations in all modular channels is equal to

$$\begin{array}{c} N_{add}=\sum_{l=2}^{k}N_{add}\left(l\right)+2\left(k-2\right)=\frac{k^{2}+3k-8}{2}. \end{array}$$

When pipelined, the throughput rate of the proposed method is one reverse conversion in one modular clock cycle.

## 7. Discussion

As it follows from [1], the calculation of the mixed-radix digits $x_{1},x_{2},\cdots ,x_{k}$ (see (6) and (7)) requires k−1 both addition and multiplication operations; in this case, the overall conversion time is $k\left(k-1\right)/2\cdot \left(t_{add}+t_{mul}\right)$, where $t_{add}$ and $t_{mul}$ denote an execution time of addition/subtraction and multiplication, respectively. The computational cost of the pipelined implementation of this algorithm is $k\left(k-1\right)/2$, both multiplication and addition operations, while the conversion time is $\left(k-1\right)\left(t_{add}+t_{mul}\right)$. The main drawback of this method is its strictly sequential nature.

The parallel conversion method circumscribed in [16] uses the additional lookup tables. At the same time, k(k+1)/2 lookup tables and k(k+1)/2 adders are required. The conversion time is $t_{lut}+\left(k-1\right)t_{add}$ due to the need to generate the inter-digit carries when performing addition operations. As noted in [34], the method proposed in [16] does not allow obtaining the claimed depth of $O\left(\log_{2}k\right)$ in terms of RNS processing elements. In this regard, an improved method was proposed by adding extra k(k+1)/2 multipliers to hardware resources used in [16]. The implementation time is $t_{lut}+t_{mul}+\left(2\log_{2}k+1\right)t_{add}$. Hence, the time complexity of this conversion algorithm is $O\left(\log_{2}k\right)$.

In [15], the mixed-radix conversion is realized by the cascaded scheme of lookup tables and adders. The computational cost for the sequential implementation is k(k−2)/4 double-size lookup tables and k(k−2)/4 adders, while the conversion time equals $\left(k/2\right)\cdot$$\left(t_{lut}+t_{add}\right)$. When pipelined, the throughput rate is determined by the time equals $t_{lut}+t_{add}$. This method works well when the used moduli do not have a very large word-length, since the size of lookup tables increases significantly with a word-length growth.

The paper [17] presents the parallel reverse conversion method, which uses the lookup table technique and requires no arithmetic or logical units. As reported, this algorithm is better than the ones presented in [15,16]. It is based on solving k(k−1)/2 linear Diophantine Equations and requires k(k−1)/2 lookup tables of size $m_{i}\times m_{j}$, while a conversion time is $\left(k-1\right)t_{lut}$. When pipelined, its effective conversion rate is one conversion per $t_{lut}$. So, this method is attractive for DSP implementation. However, it is not suitable for implementing cryptographic applications because of the enormous size of the required lookup tables, especially when processing large numbers.

In the paper [9], the reverse conversion method is based on modular reduction by a modified canonic CRT algorithm. This enables minimizing the bit-width of intermediate data processing. The lookup tables translate the $b_{i}$-bit input residues $\left(i=1,2,\cdots ,k\right)$ into $b_{out}$-bit output integers, where $b_{i}=\left\lceil \log_{2}m_{i}\right\rceil$, $b_{out}=\left\lceil \frac{1}{2}\log_{2}\left(\sum_{i=1}^{k}b_{i}\right)\right\rceil$, and *k* is the number of RNS moduli. As a result, the modular reduction of the modified *k*-tuple of $b_{out}$-bits integers is carried out over a ring of size $2b_{out}$ such that only the $b_{out}$ least significant bits of the binary representation are maintained. In this case, all the $b_{out}$-bit outputs in the modified *k*-tuple are added together by adder tree without regard to overflow, propagating the $b_{out}$ least significant bits to the output. The reverse conversion requires *k* lookup tables and k−1 adders. The scope of used lookup tables is $2^{b}\times 2^{b_{out}}$, $b\in \{b_{1},b_{2},\cdots ,b_{k}\}$. The overall conversion time is $t_{lut}+\left\lceil \log_{2}k\right\rceil \;t_{add}$.

Some reverse conversion methods use the special moduli sets with a limited number of moduli, such as $m=2^{n}+d$$\left(d\in \left\{-1,0,1\right\}\right)$ [2,8,35,36,37,38,39,40]. Their main drawback consists in a small number of the selected moduli, typically from three to five. These moduli sets are suitable for the efficient implementations of DSP algorithms but completely not applicable for large numbers processing widely used in cryptography. For example, to represent 1024-bit word-length cryptographic numbers using four RNS moduli, each modular channel must have residues of 256-bit length, which is not qualified for high-performance computing.

Table 3 compares the results across multiple techniques of the reverse conversion. Here, we use the following abbreviations: LUT—lookup table, ADD–adder, MUL—multiplier. The bit length $b\in \left\{b_{1},b_{2},\cdots ,b_{k}\right\}$, $b_{l}=\left\lceil \log_{2}m_{l}\right\rceil$$\left(l=1,2,\cdots ,k\right)$.

As seen from above, the proposed parallel reverse conversion method has time complexity of the order $O\left(\left\lceil \log_{2}k\right\rceil \right)$. In pipelined mode, it enables the high throughput rate and has one reverse conversion in one modular clock cycle. At the same time, the computational complexity is of the order of $O(k^{2}/2)$ in terms of the number of both required arithmetic operations and one-input lookup tables.

## 8. Conclusions

In this paper, a novel approach to parallel reverse conversion of the residue code $\left(\chi_{1},\chi_{2},\cdots ,\chi_{k}\right)$ of the number *X* to mixed-radix representation $\left(x_{k},x_{k-1},\cdots ,x_{1}\right)$ is described.

The calculation of the mixed-radix digits $\left(x_{k},x_{k-1},\cdots ,x_{1}\right)$ is reduced to a parallel summation of the small word-length residues $R_{1,l}\left(\chi_{1}\right)$, $R_{2,l}\left(\chi_{2}\right)$, ⋯, $R_{l,l}\left(\chi_{l}\right)$ modulo $m_{l}$ in *l*th modular channel $\left(l=1,2,\cdots ,k\right)$, taking into account the number of the overflows occuring during the modular addition operations. These modular operations are performed fast and independently in each modular channel and easily pipelined.

The computational cost of the proposed reverse conversion method is presented. In all modular channels, the general number of modular addition operations is equal to $N_{add}=\left(k^{2}+3k-8\right)/2$. At the same time, the summary number of reqiured one-input lookup tables makes up $N_{lut}=\left(k^{2}+k-2\right)/2$.

The execution time of the reverse conversion equals $T_{conv}=\left\lceil \log_{2}k\right\rceil +2$ modular clock cycles. At the same time, when pipelined, the throughput rate of the proposed conversion method is one conversion in one modular clock cycle.

The proposed parallel reverse conversion method coincides with the development vector of modern high-performance computing using residue arithmetic. It can find a widespread application for implementing a broad class of tasks in various areas of science and technology, first of all, in digital signal processing and cryptography.

## Figures and Tables

**Table 1:** The pre-calculation components.

Input Residue	Number and Skope of LUTs	Output Residue Set
$\chi_{1}$	k−1, $2^{b_{1}}\times b_{l}$ $\left(l=2,3,\cdots ,k\right)$	$\langle R_{1,2}\left(\chi_{1}\right),\;R_{1,3}\left(\chi_{1}\right),\;\cdots ,R_{1,k}\left(\chi_{1}\right)\rangle$
$\chi_{2}$	k−1, $2^{b_{2}}\times b_{l}$ $\left(l=2,3,\cdots ,k\right)$	$\langle R_{2,2}\left(\chi_{2}\right),\;R_{2,3}\left(\chi_{2}\right),\;\cdots ,R_{2,k}\left(\chi_{2}\right)\rangle$
⋯	⋯	⋯
$\chi_{k-1}$	2, $2^{b_{k-1}}\times b_{l}$ $\left(l=k-1,k\right)$	$\langle R_{k-1,k-1}\left(\chi_{k-1}\right),\;R_{k-1,k}\left(\chi_{k-1}\right)\rangle$
$\chi_{k}$	1, $2^{b_{k}}\times b_{k}$	$\langle R_{k,k}\left(\chi_{k}\right)\rangle$

**Table 2:** The results of calculations in the modular channels.

Modular Channel	Input Data	Output Data
$m_{2}$	$\langle R_{1,2}\left(\chi_{1}\right),\;R_{2,2}\left(\chi_{2}\right)\rangle$	$x_{2}^{\left(R\right)}$, $x_{3}^{\left(Q\right)}$
$m_{3}$	$\langle R_{1,3}\left(\chi_{1}\right),\;R_{2,3}\left(\chi_{2}\right),\;R_{3,3}\left(\chi_{3}\right)\rangle$	$x_{3}^{\left(R\right)}$, $x_{4}^{\left(Q\right)}$
⋯	⋯	⋯
$m_{k-1}$	$\langle R_{1,k-1}\left(\chi_{1}\right),\;R_{2,k-1}\left(\chi_{2}\right),\;\cdots ,R_{k-1,\;k-1}\left(\chi_{k-1}\right)\rangle$	$x_{k-1}^{\left(R\right)}$, $x_{k}^{\left(Q\right)}$
$m_{k}$	$\langle R_{1,k}\left(\chi_{1}\right),\;R_{2,k}\left(\chi_{2}\right),\;\cdots ,\;R_{k,k}\left(\chi_{k}\right)\rangle$	$x_{k}^{\left(R\right)}$

**Table 3:** RNS-to-MRS reverse conversion methods.

Method	Number and Scope of LUTs	ADD	MUL	Conversion Time
[1],				
sequential	–	k−1	k−1	$\frac{k\left(k-1\right)}{2}\left(t_{mul}+t_{add}\right)$
[1],				
sequential,				
pipelined	$\frac{k\left(k-1\right)}{2}$; $2^{\left(b+1\right)}\times b$	$\frac{k\left(k-1\right)}{2}$	–	$\left(k-1\right)\left(t_{lut}+t_{add}\right)$
[16],				
parallel	$\frac{k\left(k+1\right)}{2}$; $2^{b}\times b$	$\frac{k\left(k+1\right)}{2}$	–	$t_{lut}+\left(k-1\right)t_{add}$
[34],				
parallel	$\frac{k\left(k+1\right)}{2}$; $2^{b}\times b$	$\frac{k\left(k+1\right)}{2}$	$\frac{k\left(k+1\right)}{2}$	$t_{lut}+t_{mul}+\left(2\log_{2}k+1\right)t_{add}$
[15],				
sequential	$\frac{k\left(k-2\right)}{4}$; $2^{2b}\times 2b$	$\frac{k\left(k-1\right)}{4}$	–	$\frac{k}{2}\left(t_{lut}+t_{add}\right)$
[15],				
parallel	$\frac{k\left(k-2\right)}{4}+k-1$; $2^{2b}\times 2b$	$\frac{k\left(k+2\right)}{4}-3$	–	$t_{lut}+\frac{k}{2}t_{add}$
[17],				
parallel	$\frac{k\left(k-1\right)}{2}$; $2^{2b}\times b$	–	–	$\left(k-1\right)t_{lut}$
[9]	*k*; $2^{b}\times 2^{\left\lceil \frac{1}{2}\log_{2}\left(k\;b\right)\right\rceil }$	k−1	–	$t_{lut}+\left(\left\lceil \log_{2}k\right\rceil \right)t_{add}$
Our method,				
parallel	$\frac{k^{2}+k-2}{2}$; $2^{b}\times b$	$\frac{k^{2}+3k-8}{2}$	–	$\left(\left\lceil \log_{2}k\right\rceil +2\right)t_{mod}$

## Data Availability

Not applicable.
